# The food and the mood

**DOI:** 10.1080/16512235.2017.1281963

**Published:** 2017-03-15

**Authors:** Tore Midtvedt

**Affiliations:** ^a^ Department of Microbiology, Tumor and Cell Biology (MTC), Karolinska Institutet, Stockholm, Sweden

Welcome to a one-day symposium in our series ‘Science meets Industry’. As indicated in the title, the focus will be on whether and to what extent our food can influence health, especially the mood. This is indeed a topical question: in parallel with the increasing concern about so-called lifestyle diseases and syndromes, there has been an increased interest in possible preventive dietary regimens.

Today we will not focus on any regimens; we will focus on one key compound from each of our three major dietary groups, i.e. carbohydrates, protein and fat. The overall intention is to reduce the gap between basic science and the food industry.

## Carbohydrates

Beta-glucans are essential parts in many natural carbohydrates, and of specific interest are beta-glucans from oats. In former days oats were often looked upon as ‘food for the poor’, but now it is recognized that this food influences many functions in physiology, immunology and gut microbiology. Today you will learn how structures create functions, how to plan and perform a dietary study with beta-glucans as the variable compound, and how to go public with the products.

## Protein

Tryptophan is an essential amino acid to humans, and is involved in a long series of important functions in our body, e.g. as a building block in many enzymes and a precursor for important neuromodulators such as serotonin and melatonin, and it also influences immunological mechanisms. Breakdown products (e.g. kynurenines and indole)) may also play important physiological and pathophysiological roles. The importance of adequate amounts of tryptophan in our diet will be underlined. In this context, it has to be kept in mind that the most commonly used herbicide worldwide, glyphosate, acts by blocking the synthesis of tryptophan in plants and microbes, thus influencing our natural supply of this essential amino acid. The lecturer has a lifelong experience in working with various aspects regarding dietary tryptophan.

## Fat

For decades, fat was looked upon as the ‘bad guy’ in our diet. Now it is included in some specific dietary regimens, e.g. the low carbohydrate/high fat diet, and the importance is well recognized of some fatty acids, such as omega-3 and omega-6 in immunological homeostasis. The lecturer in this symposium will focus upon a completely new field: omega-3 and omega-6 as regulators of mood. I am sure that the state-of-art will be outlined, and the possible mechanisms behind their effects on mental status will be presented and discussed.

Taken together, these three key dietary elements have important roles to play, in physiology as well as in pathophysiology. Whether you are a scientist, in industry or a regulatory person, or just a consumer, you will get some new information of importance for your daily life and work!

## Concluding remarks

### What did we learn in the symposium today?

#### Tore Midtvetd

I think that we all can agree that there are huge amounts of new basic scientific information regarding physiological and pathophysiological roles of beta-glucans, tryptophan, omega-3 and omega-6. I also think that we all have recognized the willingness of the lecturers to go public with their knowledge. I have also recognized that representatives from industry and food agencies have been eagerly listening and have taken notes. Simply spoken, they ‘mind the gap’ between science, industry and consumers and that was the main idea behind this symposium. I believe that this type of meetings is here to stay. Thanks for coming and welcome back!

